# A new species of sole (Pleuronectiformes, Soleidae), *Soleichthys labyrinthus*, from the China Sea

**DOI:** 10.3897/zookeys.1294.197188

**Published:** 2026-10-12

**Authors:** Junjie Zheng, Zhiqi Zou, Siyuan Li, Yang He, Jiajie Chen, Hanye Zhang, Mingru Chen, Xiaoyu Kong

**Affiliations:** 1 State Key Laboratory of Marine Environmental Science, and College of Ocean and Earth Sciences, Xiamen University, Xiamen, 361102, China State Key Laboratory of Marine Environmental Science, and College of Ocean and Earth Sciences, Xiamen University Xiamen China; 2 School of Advanced Interdisciplinary Biomedical Sciences, Xiamen University, Xiamen, 361102, China School of Advanced Interdisciplinary Biomedical Sciences, Xiamen University Xiamen China; 3 East China Sea Fisheries Research Institute, Chinese Academy of Fishery Sciences, Shanghai, 200090, China East China Sea Fisheries Research Institute, Chinese Academy of Fishery Sciences Shanghai China; 4 Key Laboratory of Fisheries Remote Sensing, Ministry of Agriculture and Rural Affairs, Shanghai, China Key Laboratory of Fisheries Remote Sensing, Ministry of Agriculture and Rural Affairs Shanghai China; 5 Fujian Institute for Sustainable Oceans, Xiamen University, Xiamen, 361102, China Fujian Institute for Sustainable Oceans, Xiamen University Xiamen China; 6 Guangdong Provincial Key Laboratory of Applied Marine Biology, South China Sea Institute of Oceanology, Chinese Academy of Science, Guangzhou, Guangdong Province, 510301, China Guangdong Provincial Key Laboratory of Applied Marine Biology, South China Sea Institute of Oceanology, Chinese Academy of Science Guangzhou China

**Keywords:** Fish taxonomy, flatfish, key, marine fish, morphology, phylogeny

## Abstract

The genus *Soleichthys* (Soleidae) suffers from taxonomic confusion due to morphological similarities. In this study, a new sole species, *Soleichthys labyrinthus***sp. nov**., is described based on the holotype and 24 paratypes collected from the northern South China Sea and the East China Sea. This new species is readily distinguished from its congeners by a unique combination of characters: 78–86 dorsal-fin rays and 65–73 anal-fin rays; the ocular side is tawny brown, featuring a labyrinth-shaped reticulated pattern and lacking the typical transverse bands found in most congeners. The ocular-side lateral line possesses two prominent white spots, accompanied by a series of four (occasionally five) small, longitudinally arranged flaxen-white to light-brown spots occurring along both the dorsal-fin base and the anal-fin base. The caudal fin also bears a broad black transverse band followed posteriorly by a broad white marginal band. Phylogenetic analysis based on the mitochondrial cytochrome *c* oxidase subunit I (*COI*) gene robustly supports *S. labyrinthus***sp. nov**. as a distinct monophyletic lineage, while K2P genetic distances reveal deep interspecific divergence (0.182–0.227) from its congeners. This study also provides an updated taxonomic key to all eleven species of *Soleichthys*.

## Introduction

*Soleichthys* Bleeker, 1860 is a genus of the family Soleidae. Morphologically, the genus differs from other soleid genera by having an extremely elongated tubular anterior nostril on the ocular side, which, when directed posteriorly, can reach the lower orbit or even extend beyond the middle of the lower orbit (Wu and Zhong 2021). Certain species can alter their body coloration in response to environmental cues, making them highly adept at camouflage (Wu and Zhong 2021), which further complicates morphology-based species identification of *Soleichthys*.

The genus *Soleichthys* has undergone multiple taxonomic revisions. Bleeker (1860) transferred *Solea heterorhinos* to *Soleichthys heterorhinos*, described in 1856, to his newly erected genus *Soleichthys*, designating it as the type species. Günther (1862) reassigned *S. heterorhinos* back to *Solea*. *Soleichthys* was subsequently resurrected by Evermann and Seale (1907) for *S. heterorhinos*, a taxonomic arrangement adopted by numerous later authors. At present, ten valid species are generally recognized within *Soleichthys* according to Eschmeyer’s Catalog of Fishes (Fricke et al. 2026): *S. dori* Randall & Munroe, 2008; *S. heterorhinos* (Bleeker, 1856); *S. maculosus* Muchhala & Munroe, 2004; *S. microcephalus* (Günther, 1862); *S. multifasciatus* (Kaup, 1858); *S. nigrostriolatus* (Steindachner & Kner, 1870); *S. oculofasciatus* Munroe & Menke, 2004; *S. serpenpellis* Munroe & Menke, 2004; *S. tubiferus* (Peters, 1876) and *S. siammakuti* Wongratana, 1975. To date, records of *Soleichthys* from Chinese waters remain limited. *Soleichthys heterorhinos* occurs in the East China Sea and the South China Sea (Liu et al. 2025), whereas *S. tubiferus* has been reported from Weizhou Island in the northern South China Sea (Luo et al. 2025). An undescribed *Soleichthys* specimen was recorded from the waters off Taiwan, China (Shen and Lee 1981). In addition, *S. siammakuti* Wongratana, 1975 has been listed from the South China Sea (Randall and Lim 2000), but its occurrence in Chinese waters requires confirmation, as recent specimen-based records are from Malaysian and Philippine waters (Matsunuma et al. 2024).

In early 2026, during fish sampling at a fish market along the northern coast of the South China Sea, we collected several specimens assignable to *Soleichthys* based on well-developed pectoral fins on both sides and extremely elongated anterior nostrils. These specimens could be distinguished from other congeners and the *Soleichthys* sp. from Taiwan, China, by the unique flaxen-white labyrinth-shaped colour pattern on the ocular side. The present paper provides a taxonomic description of this new species and an updated taxonomic key to all eleven recognized species of *Soleichthys*.

## Material and methods

### Sample collection and preservation

A total of 25 specimens were collected. Of these, 23 were obtained from a fish market in Xiamen, Fujian, China. According to local fishermen, these individuals were captured by bottom trawling in the northern South China Sea in March and April 2026. Two additional specimens were collected by bottom trawl from the East China Sea in September 2025 and December 2022.

Specimens examined in this study are catalogued and deposited in fish collections of **ECSFRI** (East China Sea Fisheries Research Institute, Chinese Academy of Fishery Sciences, Shanghai, China) and **IOZ** (Institute of Zoology, Chinese Academy of Sciences, Beijing, China), **SCSMBC** (South China Sea Institute of Oceanology, Chinese Academy of Science, Guangdong, China), **SNHM** (Shanghai Natural History Museum, Shanghai, China).

### Counts and measurements

**Figure 1.**
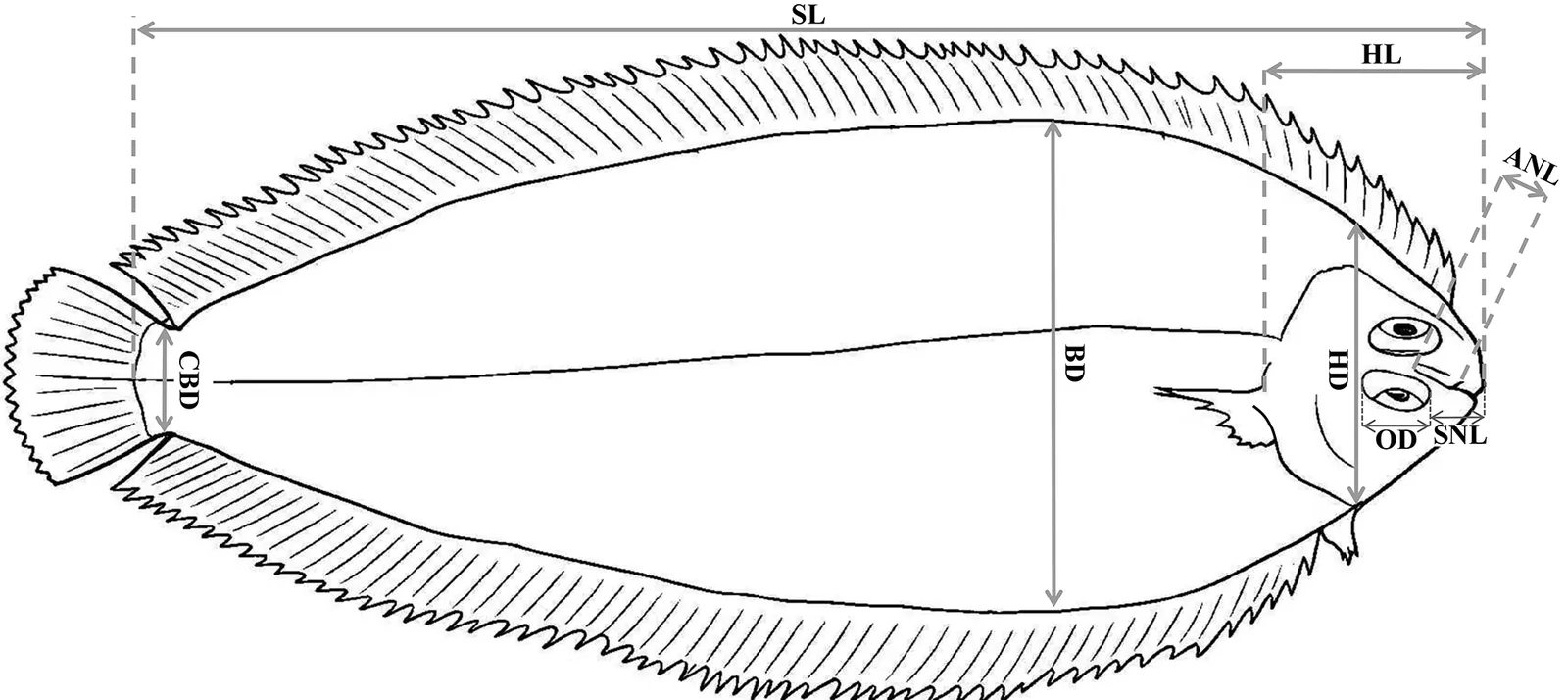
Morphological measurement illustration (ocular side). Abbreviations: SL = standard length, ANL = anterior nostril length, BD = body depth, CBD = caudal-fin base depth, HD = head depth, HL = head length, OD = orbit diameter, SNL = snout length.

Methods for counting meristic and measuring morphometric features generally follow Munroe and Menke (2004), unless otherwise specified. Standard length (**SL**); caudal-fin length (**CFL**); head length (**HL**), greatest horizontal distance from snout tip to posterior margin of operculum; snout length (**SNL**); upper-jaw length (**UJL**); head depth (**HD**), from the ventralmost point of the operculum vertically to the dorsal margin of the head; body depth (**BD**); caudal-fin base depth (**CBD**); predorsal length (**PDL**), from snout tip to dorsal-fin origin; orbit diameter (**OD**), greatest horizontal diameter of the lower orbit; anterior nostril length (**ANL**), length of anterior nostril on the ocular side; prepelvic length (**PPVL**), from snout tip to pelvic-fin origin; preanal length (**PAL**), from snout tip to anal-fin origin (Fig. 1).

**Figure 2.**
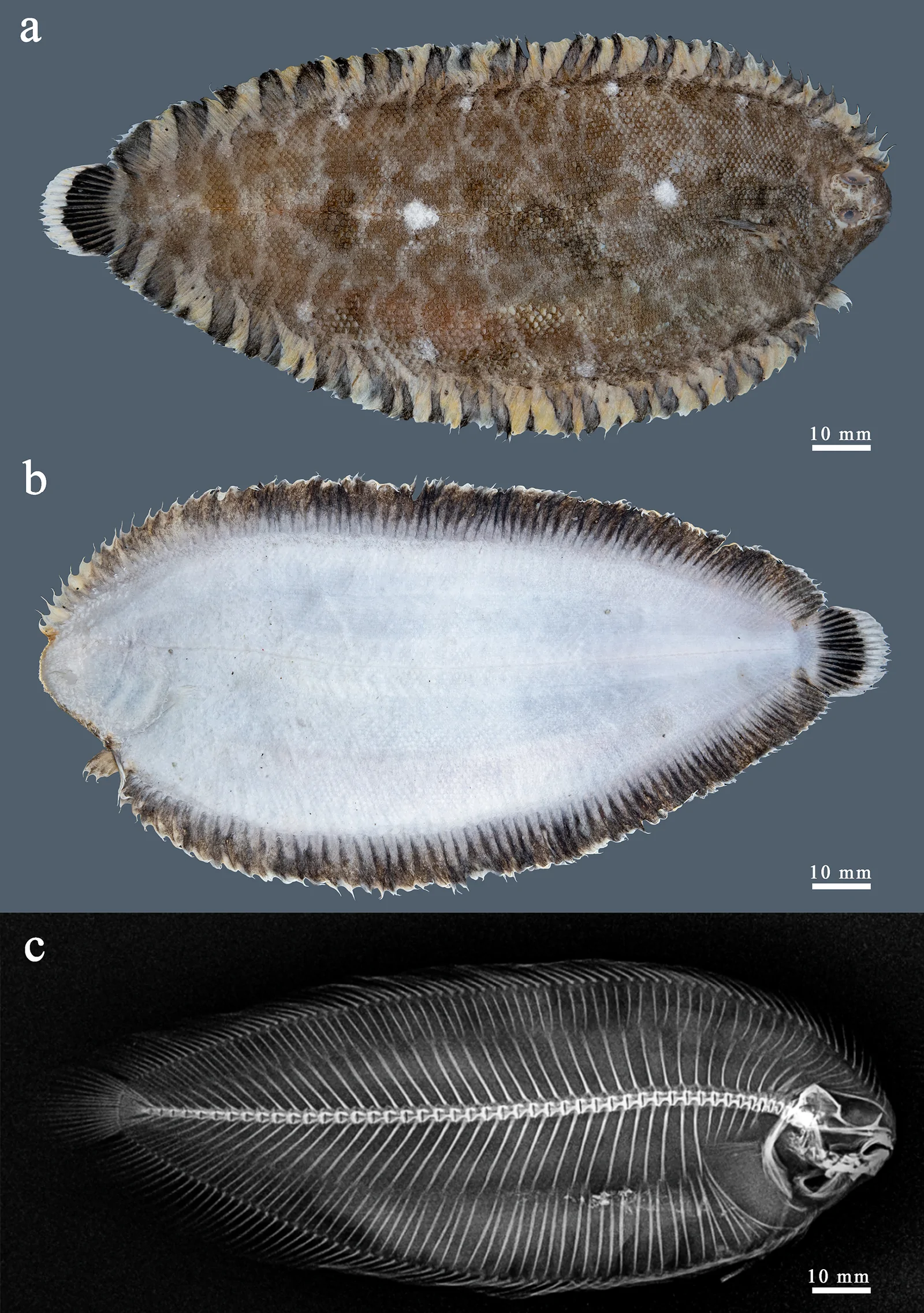
*Soleichthys labyrinthus* sp. nov., SCSMBC 110075, holotype, 134 mm SL, fresh. **a**. Ocular side; **b**. Blind side; **c**. Radiograph.

All measurements, made to the nearest 0.1 mm with vernier calipers, were taken on the ocular side except where noted. Measurements are presented as proportions of SL. Fine morphological traits, including scale structure and fin-ray branching patterns, were examined under a stereomicroscope, and trunk lateral-line scales were counted under the same microscope; vertebral counts were obtained from radiographs of the holotype (Fig. 2c) and 10 paratypes.

### Phylogenetic analysis

To determine the systematic position of the new species, *COI* barcoding sequences were analyzed. This study included 16 sequences for molecular analyses. Three were newly generated sequences (GenBank accession no. PZ955454, PZ958491, PZ962741). The remaining 13 sequences representing five species (*S. maculosus*, *S. oculofasciatus*, *S. microcephalus*, *S. heterorhinos* and *Zebrias quagga*) were retrieved from GenBank, with the species *Z. quagga* (Kaup, 1858) designated as the outgroup.

The best-fit model was selected, and the maximum-likelihood (ML) phylogeny was inferred using IQ-TREE (Nguyen et al. 2015). Nodal support was assessed using 1000 standard bootstrap replicates. The resulting phylogenetic tree was visualized and polished using Interactive Tree of Life (iTOL) (Letunic and Bork 2024). Pairwise genetic distances based on the Kimura 2-parameter (K2P) (Kimura 1980) model were calculated from the *COI* sequences using MEGA 12 (Kumar et al. 2024).

## Taxonomy

### Soleichthys labyrinthus sp. nov.

Taxon classificationAnimaliaPleuronectiformesSoleidae

D656431D-748B-5AA9-96CC-773EC0C7B9B7

https://zoobank.org/46E1787D-7EA7-475D-B967-2EABDB10E4CB

Figs 2, 3; Table 1

#### English name.

Mesh sole.

**Figure 3.**
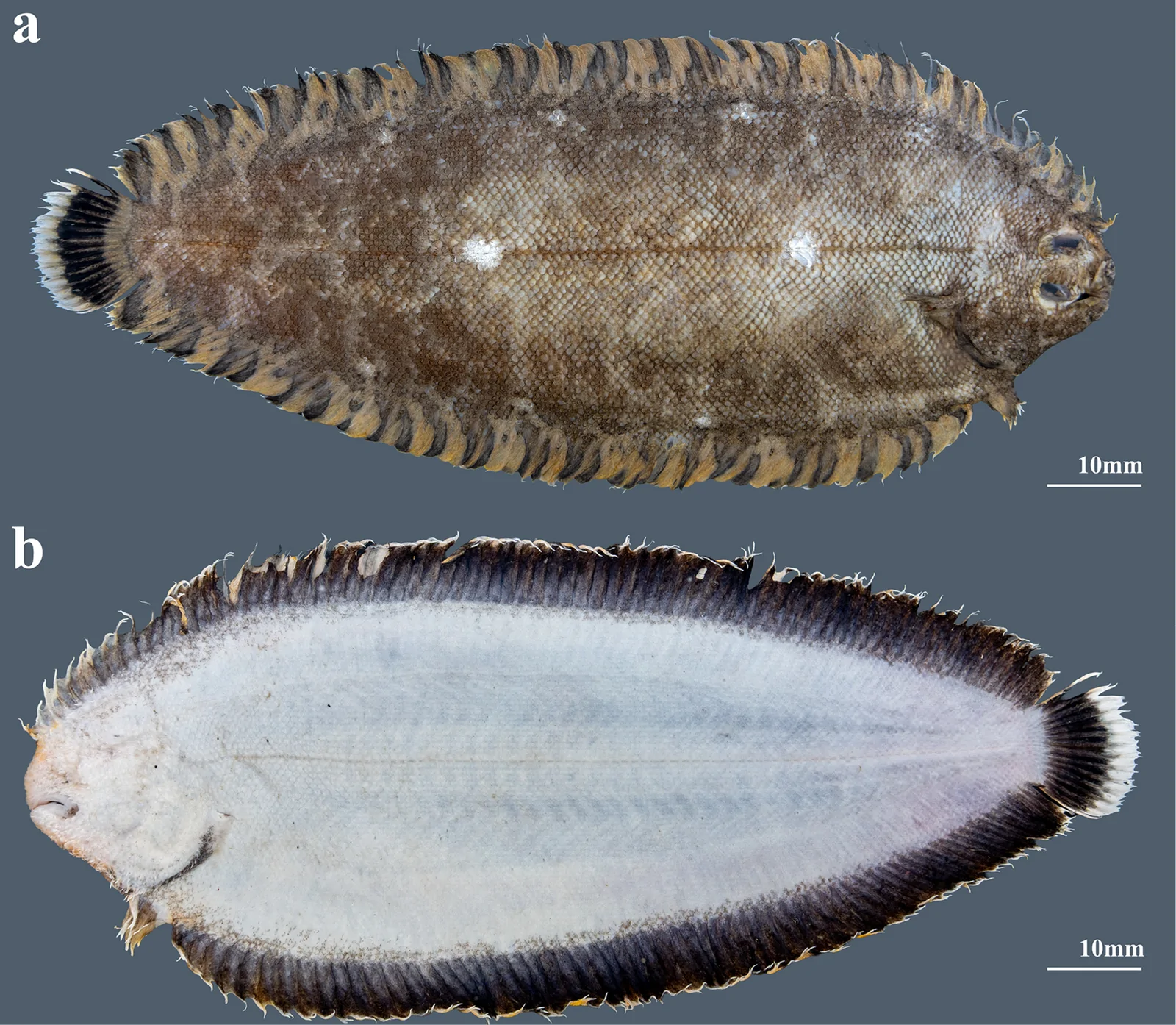
*Soleichthys labyrinthus* sp. nov., SCSMBC 110076, paratype, 127 mm SL, fresh. **a**. Ocular side; **b**. Blind side.

**Table 1.** Morphological comparison of all valid species in the genus *Soleichthys*.

	***S. labyrinthus* sp. nov**.		***S. dori***	***S. siammakuti***	***S. heterorhinos***	***S. microcephalus***	***S. oculofasciatus***	***S. serpenpellis***	***S. maculosus***	***S. tubiferus***	***S. multifasciatus***	***S. nigrostriolatus***
	**Holotype**	**Paratype**										
SL (mm)	134	96–150	39.5–106	69.7–82.4	76–126	113.4–172	107.7–124.6	100.0–125.3	71.2–95.1		119	68–127
DFR	80	78–86	87–95	73–80	92–102	71–84	79–81	76–84	78–84	77–79	95	90–92
AFR	65	65–73	77–83	62–68	77–87	61–71	68	64–71	64–69	66–70	80	78–82
PVFR	5	5	5	5	4	5	5	5	5	4	4	4–5
CFR	18	16–18	17–19	18–19	16–18	16–18	18	18–19	18			
PFR (O)	9	8–10	8–10	8	7–9	8–11	11	8–9	8–9	9–10	7–10	8–9
PFR (B)	7	6–8	7–9	7–8	6–8	7–11	10–11	6–8	7–9	7		
LLS	85	84–96	98–115	88–108	99–122	81–89	85–86	83–95	90–100	94–102	123	99–110
Vertebrae	45	43–45	50–51	44	51	43–45	45–46	44–46	45–46	45		
	9 + 36	8–9 + 35–37		9 + 35	8 + 42					9 + 36		
% SL												
BD	37.4	32.2–37.5	30.5–32.6	32.4–37.3	39.0–41.0	35.7–44.6						
HL	16.6	15.0–18.9	17.7–22.5	16.7–19.9	16.6–17.3	16.0–18.1	36.2–36.3	35.0–38.2	35.3–36.8	38.5		
HD	20.9	18.5–23.4	20.9–22.7	20.3–25.5	21.4–22.7	19.8–25.6	18.5–19.1	15.3–17.7	17.4–19.4	17.5		
SNL	4.9	4.2–5.9	4.6–5.5	3.9–4.4	3.7–4.0	2.9–4.7	19.1–19.3					
OD	3.3	2.5–4.2	3.2–5.1	2.9–5.1	3.3–3.5	2.8–4.1	3.7–4.0	3.2–4.7				
ANL	3.1	2.8–3.9	6.3–8.4	3.8–6.2	4.9		3.7–3.8	3.3–4.7				
UJL	5.3	4.6–6.0	5–7.3	5.1	4.4–6.3	2.8–4.4						
CBD	6.7	5.3–8.0	5.3–5.7	6.9–8.5	6.0–6.7		4.3–4.4	3.5–4.7				
PDL	4.9	2.6–5.7	6–7	7.4–7.5	2.4–2.6	1.9–3.8						
PAL	17.4	17.3–20.6	16.2–17.9	18.4–20.8	14.3–16.2		2.2–2.4	2.3–2.7				
PPVL	13.9	11.6–15.8	13–13.8	14.1–14.6	9.9							
LDR	7.5	5.6–8.9	11.9–14.2	9.1–9.7	9.4–10.3							
LAR	7.5	6.0–8.5	12.3–14.7	10.9–11.2	10.0–10.2							
LPR (O)	8.3	6.4–9.4		8.7	3.4–5.6	7.8–12.2						
LPR (B)	2	1.9–3.4		2.4–3.4	1.6–3.0		10.2	10.6–16.9				
CFL	9.5	9.3–12.5	13.9–16.8	14.8–16.0	12.1–12.8	12.6–14.5						
PVFL	6.2	3.5–6.8	6.6–7.8	4.7–6.1	5.8		14.9–16.8	12.6–14.0				

Note: DFR, dorsal-fin ray; AFR, anal-fin ray; PVFR, pelvic-fin ray; CFR, caudal-fin ray; PFR (O), pectoral-fin ray (ocular side); PFR (B), pectoral-fin rays (blind side); LLS, lateral-line scale; LDR, longest dorsal ray; LAR, longest anal ray; LPR (O), longest pectoral ray (ocular side); LPR (B), longest pectoral ray (blind side); CFL, caudal-fin length; PVFL, pelvic-fin length. Data for *S. heterorhinos* are based on specimens examined in the present study and previously published data (Li and Wang 1995; Nakabo 2013; Ohashi and Motomura 2011). Data for other congeners are compiled from the literature (Heemstra et al. 2022; Matsunuma et al. 2024).

#### Chinese name.

迷网长鼻鳎 (Míwăng Chángbítă).

#### Etymology.

The specific epithet is derived from the Latin *labyrinthus*, meaning labyrinth-shaped. It refers to the intricate flaxen-white slim stripes on the dark brown surface of the ocular side of this species.

#### Holotype.

• SCSMBC 110075, 134 mm SL, China, purchased at a fish market in Xiamen, Fujian, China, 15 March 2026, reportedly collected in the northern South China Sea.

#### Paratypes.

All specimens were collected at the same locality and in the same manner as the holotype, unless otherwise stated. • SCSMBC 110076, 127 mm SL; • SCSMBC 110077–110081 were collected at the same locality on 21 March 2026: SCSMBC 110077, 146 mm SL; SCSMBC 110078, 143 mm SL; SCSMBC 110079, 141 mm SL; SCSMBC 110080, 114 mm SL; SCSMBC 110081, 134 mm SL; • SNHM-Hfi 13609–13611 were collected at the same locality on 22 March 2026: SNHM-Hfi 13609, 140 mm SL; SNHM-Hfi 13610, 141 mm SL; SNHM-Hfi 13611, 134 mm SL; SNHM-Hfi 13612, 134 mm SL, 24 March 2026; SNHM-Hfi 13613, 122 mm SL, 26 March 2026; • ECSFRI 35001–35005 were collected at the same locality on 3 April 2026: ECSFRI 35001, 112 mm SL; ECSFRI 35002, 128 mm SL; ECSFRI 35003, 128 mm SL; ECSFRI 35004, 142 mm SL; ECSFRI 35005, 137 mm SL, 12 April 2026; ECSFRI 30129, 96 mm SL, December 2022, East China Sea, Fish Ground 303, Dongshan; • IOZ 280001–280006 were collected at the same locality on 4 April 2026: IOZ 280001, 123 mm SL; IOZ 280002, 136 mm SL; IOZ 280003, 141 mm SL; IOZ 280004, 130 mm SL; IOZ 280005, 125 mm SL; SCSMBC 113023, 150 mm SL, Quanzhou, Fujian, China, September 2025.

#### Diagnosis.

*Soleichthys labyrinthus* sp. nov. is distinguished from its congeners by the following combination of characters: dorsal-fin rays 78–86, anal-fin rays 65–73; pelvic-fin rays 5 on each side; lateral-line scales 84–96; total vertebrae 8–9 + 35–37 = 43–45. The 2–3 uppermost rays of the ocular-side pectoral fin are subequal in length. The basal one-third of the 3–4 uppermost pectoral-fin rays is covered with ctenoid scales. The ocular side is tawny brown, without transverse bands or vermiculate stripes, and is interspersed with irregular flaxen-white stripes forming a labyrinth-shaped reticulated pattern. Two white blotches are arranged anteriorly and posteriorly on the lateral line. The caudal fin has a broad black band followed by a broad white marginal band. The dorsal and anal fins on the blind side are dark brown to black, with white ray tips and fin bases.

#### Description.

**Figure 4.**
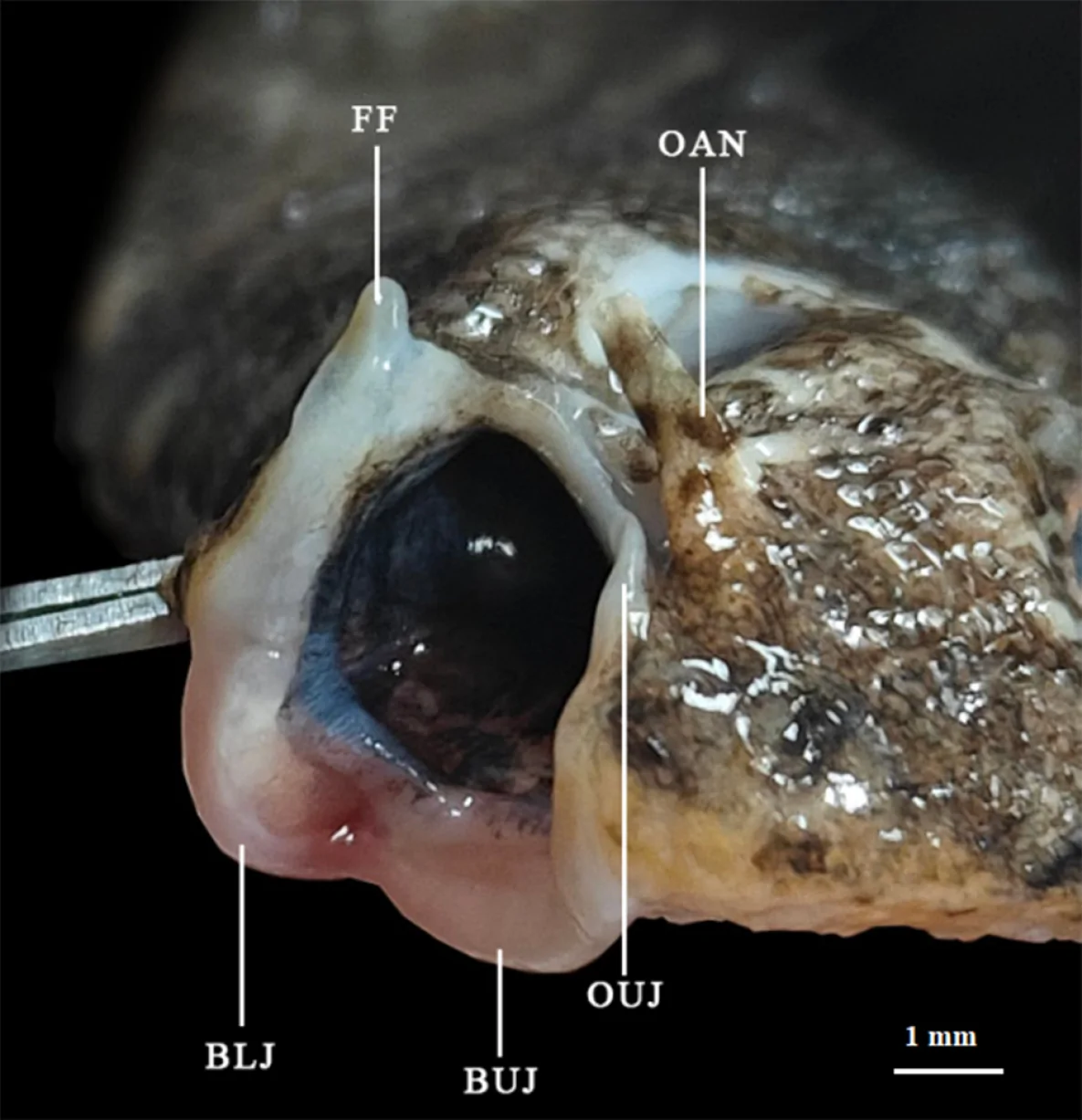
Anterior view of head of paratype SNHM-Hfi 13611. Abbreviations: FF = fleshy flap, OAN = ocular-side anterior nostril, BLJ = blind-side lower jaw, BUJ = blind-side upper jaw, OUJ = ocular-side upper jaw.

Meristic and morphometric features of the holotype and 24 paratypes, together with comparisons with ten congeners, are summarized in Table 1. Body moderately elongate, strongly compressed. Eyes dextral, about equal in size and slightly smaller than SNL. Interorbital space narrow. Snout blunt and short; mouth small, terminal to slightly subinferior; maxilla reaches to level below anterior part of lower eye. On the ocular side, upper and lower jaws slender; lower lip bears a free, fleshy quadrangular dermal process at the midpoint (Fig. 4). Ocular-side opercular margin lacks cirri; blind-side margin bears numerous short fleshy cirri around the gill opening. No dermal papillae on the ocular-side head; papillae and fleshy cirri occur on the blind-side snout and anterior head beside the dorsal-fin base. Ocular-side jaws are toothless, while the blind side bears a single row of slender conical teeth on each jaw. Gill openings on the ocular and blind sides are united ventrally at the isthmus; their posterior margins are free from the isthmus.

There are two nostrils on the ocular side. The anterior nostril is slender tubular, elongated, reaching the anterior margin of the lower eye or nearly to its midpoint when directed posteriorly. The ocular-side posterior nostril is short, broad and ventrally located just above the upper jaw. There are two nostrils on the blind side; anterior nostril is a slightly elevated, short, wide tube located dorsal to the mid-jaw region. The posterior blind-side nostril is short, posteriorly directed tube.

Scales are ctenoid except for those of the lateral line on both sides. On the ocular side, each ctenoid scale bears up to 12 sharp cteni. Scales on the fin bases and head gradually become smaller laterally, with the number of cteni reduced to 6–8. Scales on the anterior head become short, fleshy, pointed papillae. The lateral line extends anteriorly in a straight line following the midline of the body. Above the base of the pectoral fin, it curves dorsally for approximately 10 scale rows, then bends anteriorly and passes less than one eye diameter above the upper eye. The blind-side lateral line follows a trajectory similar to that of the ocular-side. At the posterior end of the head, it splits into several short rows of cirri. These cirri extend posteriorly as a single row onto the fin membrane between adjacent dorsal-fin rays. A broad band of small scales covers approximately the basal fourth of the median fins on the ocular side, whereas all fins on the blind side are entirely scaleless.

All fin rays distally bifurcate, except those of the pectoral fins and a few rays on each side of the caudal fin. The dorsal-fin origin is slightly shifted toward the blind side. The pelvic fins originate below the posterior margin of the lower eye, anteroventral to the pectoral-fin origin; the anal fin originates posterior to the anus, immediately behind the pelvic-fin insertion. The caudal fin is membranously connected at its extreme base to dorsal and anal fins. Dorsal- and anal-fin rays are nearly uniform in length, with the longest rays occurring at mid-body or along the posterior-to-midbody region. The uppermost two to three rays are notably elongated and subequal in length, whereas the next ray is slightly shorter; the remaining lower rays are much shorter, forming a fan-shaped arrangement, and translucent white. The basal one-third of the uppermost 3–4 pectoral-fin rays is covered with ctenoid scales. The dorsal skin of the ocular-side pectoral-fin rays is fused to the surface of the posterior operculum. The blind-side pectoral fin is fan-shaped, white, and covered with thickened skin.

#### Coloration.

The inner surface of the operculum is pale basally, with the gill cavity dotted with fine black spots. The buccal cavity is entirely black. The tubular anterior nostril is brown-spotted, except for its pale tip and inner base. The ocular side of the body is tawny brown, and patterned with narrow flaxen-white stripes, forming a distinct reticulate pattern. Along the bases of dorsal and anal fins, 4–5 longitudinally arranged pale-brown to white blotches (occasionally indistinct) are present on each fin. Two conspicuous white blotches occur on the ocular-side lateral line, one anterior and one posterior.

The dorsal and anal fins display alternating grayish orange-yellow and tawny brown transverse bands; the margins of the fin membranes are yellow to orange, with white coloration restricted to the distal tips of fin rays. The blind-side body is pale overall, whereas the dorsal and anal fins are dark brown, with white fin-ray tips. On both the ocular and blind sides, the caudal fin has a broad black transverse band, followed posteriorly by a broad marginal white band that is approximately half as wide as the black band; the proximal margin of the white band is occasionally pale yellowish.

#### Phylogenetic analysis.

**Figure 5.**
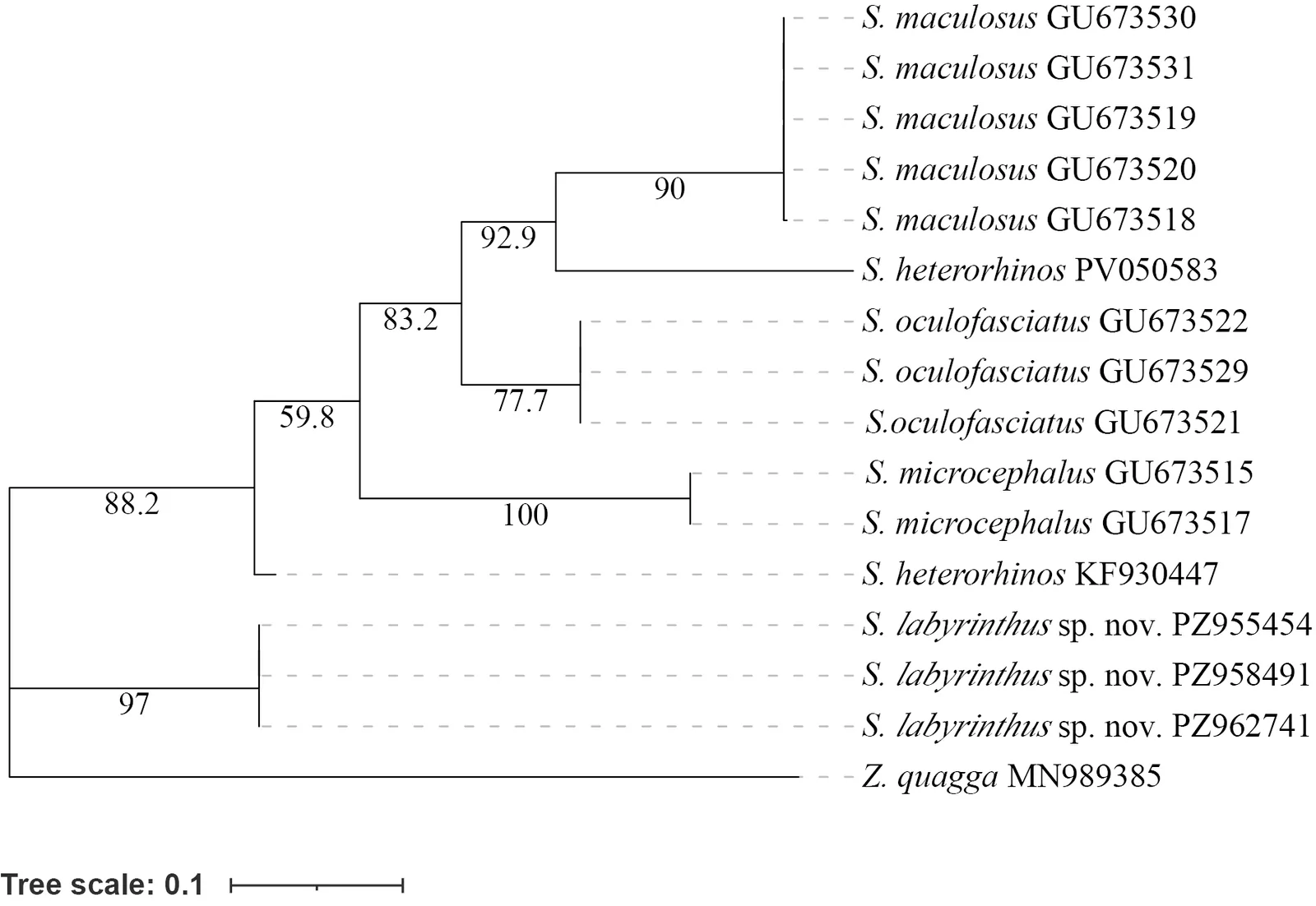
Maximum-likelihood phylogenetic tree of five species from the genus *Soleichthys* with one species as outgroup based on *COI* sequences.

Phylogenetic analysis (Fig. 5) revealed that the three specimens of *S. labyrinthus* sp. nov. formed a highly supported monophyletic clade (bootstrap = 97) and were sister to the remaining sampled *Soleichthys* species. Meanwhile, the monophyly of *S. microcephalus*, *S. maculosus* and *S. oculofasciatus* was also supported by relatively high bootstrap values. In contrast, the two database sequences identified as *S. heterorhinos* showed markedly discordant topological positions and did not form a monophyletic group, with a relatively high intraspecific K2P genetic distance (0.182) (Table 2). Therefore, the anomalous phylogenetic position of *S. heterorhinos* requires further verification.

**Table 2.** Analysis of the intraspecific and interspecific distances based on the *COI* between *S. labyrinthus* sp. nov. and closely related species.

**Species**		**Interspecific Mean Distance**					
		**1**	**2**	**3**	**4**	**5**	**6**
1	*S. labyrinthus***sp. nov**.	0.000					
2	*S. heterorhinos*	0.182–0.226	0.182				
3	*S. maculosus*	0.224–0.227	0.181–0.184	0.000–0.004			
4	*S. microcephalus*	0.205–0.208	0.169–0.221	0.182–0.187	0.002		
5	*S. oculofasciatus*	0.208	0.148–0.167	0.169–0.171	0.176	0.000	
6	*Z. quagga*	0.204	0.241–0.245	0.242	0.221–0.224	0.247	0.000

Based on *COI* data, the K2P genetic distances (Table 2) indicate a deep evolutionary divergence between *S. labyrinthus* sp. nov. and other valid species in the genus. The intraspecific genetic distances showed very low variation, ranging from 0.000 to 0.004, except for *S. heterorhinos*. Interspecific genetic distances were substantially higher, ranging from 0.182 between *S. labyrinthus* sp. nov. and *S. heterorhinos* to 0.227 between *S. labyrinthus* sp. nov. and *S. maculosus*. These results support the recognition of *S. labyrinthus* sp. nov. as a distinct species.

## Discussion

Based on morphological and phylogenetic evidence, a new species, *Soleichthys labyrinthus* sp. nov., is described from the northern South China Sea and the East China Sea. It is distinguished from *S. dori*, *S. heterorhinos*, *S. nigrostriolatus*, and *S. multifasciatus* by having 78–86 dorsal-fin rays (vs. 87–102) and 65–73 anal-fin rays (vs. 77–87 in *S. dori*, *S. heterorhinos*, and *S. nigrostriolatus*). It can also be distinguished from *S. tubiferus* and *S. multifasciatus* by having five pelvic-fin rays on each side, compared with four in *S. tubiferus* and *S. multifasciatus*. The new species has 84–96 lateral-line scales, compared with 99–122 in *S. heterorhinos* and 99–110 in *S. nigrostriolatus*, and has 43–45 vertebrae, compared with 50–51 in *S. dori* and 51 in *S. heterorhinos*.

*Soleichthys labyrinthus* sp. nov. is further distinguished from its congeners by the morphology of the ocular-side pectoral fin. The two uppermost rays are subequal in length, whereas the first ray is distinctly longer than the second in *S. oculofasciatus* and *S. siammakuti*. The basal third of the 3–4 uppermost ocular side pectoral-fin rays is covered with scales. In contrast, the ocular-side pectoral fin is completely naked in *S. maculosus* and *S. siammakuti*; scales extend to approximately mid-ray length in *S. microcephalus*; and in *S. dori*, the basal three-quarters of the ocular-side pectoral fin are scaled, except for the two slender uppermost rays, which are scaled only on their basal quarter.

Pigmentation provides conspicuous characters for distinguishing the new species from its congeners. The ocular side is tawny brown, darker along the mid-axis, with irregular flaxen-white stripes forming a labyrinth-shaped pattern; complete transverse bands or vermiculate transverse stripes are absent. Complete transverse bands occur in *S. dori*, *S. heterorhinos*, *S. siammakuti*, *S. oculofasciatus*, *S. microcephalus* and *S. multifasciatus*, while narrow, black vermiculate transverse stripes characterize *S. nigrostriolatus*. Two white blotches are present along the lateral line, which are absent in *S. tubiferus*, *S. heterorhinos*, *S. nigrostriolatus*, and *S. maculosus*. Four, occasionally five, small white to pale-brown blotches are present at the bases of the dorsal and anal fins, with the first anal-fin blotch usually opposite the second dorsal-fin blotch. These blotches and their arrangement are absent in all other congeners, except *S. oculofasciatus*, which has only two blotches at the dorsal- and anal-fin bases, respectively. The caudal fin bears a broad black transverse band followed posteriorly by a broad white marginal band. In contrast, the marginal band is narrow and white in *S. tubiferus* and black in *S. heterorhinos*, *S. dori*, *S. serpenpellis*, *S. oculofasciatus*, and *S. nigrostriolatus*.

Based on original species descriptions, relevant literature (see Table 1 note) and two non-type specimens of *Soleichthys heterorhinos*, we constructed an identification key to all 11 species of *Soleichthys*.

### Key to the species of the genus *Soleichthys*

**Table:** 

1	Ocular side without complete transverse bands and narrow, black, vermiculate transverse stripes, with irregular blotches or rhomboid bands	**2**
–	Ocular side with complete transverse bands or narrow, black, vermiculate transverse stripes	**5**
2	Caudal fin on ocular side with white proximal margin	**3**
–	Outer margin of caudal fin on ocular side not white	**4**
3	White marginal band on caudal fin of ocular side extremely narrow, much narrower than the broad black caudal-fin band; no white spots on lateral line	***S. tubiferus***
–	White marginal band on caudal fin of ocular side relatively wide, its width approximately half that of the broad black caudal-fin band; two white spots present on lateral line	***S. labyrinthus* sp. nov**.
4	Ocular side with scattered white spots and blotches, without accompanying transverse bands	***S. maculosus***
–	Transverse bands on ocular side incomplete, rhomboid-shaped, broad at centre and narrow at both ends	***S. serpenpellis***
5	Ocular side of body with transverse bands; anal and caudal fins with large, bright yellow spots when fresh, appearing as semitransparent spots in preserved specimens	***S. siammakuti***
–	Ocular-side dorsal, anal, and caudal fins relatively uniformly colored, without round spots	**6**
6	Transverse bands on ocular side light-coloured; interspace between bands usually wider than the bands themselves	**7**
–	Transverse bands on ocular side dark brown, or consisting of narrow, black vermiculate transverse stripes	**8**
7	Two elongate ocular-side pectoral-fin rays, with the first ray noticeably longer than the second; scales absent on the base and proximal halves of the elongate ocular-side pectoral-fin rays	***S. oculofasciatus***
–	Two elongated ocular-side pectoral-fin rays nearly equal in length, or second ray longer than first; small scales present on the bases and proximal halves of the elongated ocular-side pectoral-fin rays	***S. microcephalus***
8	Ocular side with slightly wavy, double, dark brown bands across body, becoming irregular posteriorly	**9**
–	Ocular side with numerous narrow, black vermiculate transverse stripes; markings highly irregular in arrangement, often fragmented into black spots	***S. nigrostriolatus***
9	Lateral-line scales less than 122; ocular-side body with about 16 relatively evenly spaced, narrow, dark-brown transverse bands	**10**
–	lateral-line scales 123; ocular-side body with about 27 narrow transverse bands	***S. multifasciatus***
10	Snout length greater than eye diameter; white spots present on lateral line of ocular side; ocular-side body with about 16 transverse bands	***S. dori***
–	Snout length less than eye diameter; no white spots on lateral line of ocular side	***S. heterorhinos***

### Comparative materials

*Soleichthys heterorhinos*SCSMBC 113025, 112 mm SL; SCSMBC 113024, 107 mm SL.

## Supplementary Material

XML Treatment for Soleichthys labyrinthus

## Additional information

### Artificial Intelligence (AI) use

The authors accept full responsibility for the content of the manuscript, including the disclosure of any use of AI.

No AI tools were used in the preparation of this manuscript.

### Author ORCIDs

J. Zheng https://orcid.org/0009-0004-3705-4130

Z. Zou https://orcid.org/0009-0004-1357-5265

S. Li https://orcid.org/0009-0008-5413-7797

Y. He https://orcid.org/0009-0009-1700-2751

J. Chen https://orcid.org/0000-0001-7487-231X

H. Zhang https://orcid.org/0000-0002-1645-6796

M. Chen https://orcid.org/0000-0001-5295-0196

X. Kong https://orcid.org/0000-0003-0010-7425

### Data availability

All of the data that support the findings of this study are available in the main text.
